# Applying the Somatic Symptom Disorder Diagnosis to Individuals with Fibromyalgia: Strengths and Limitations

**DOI:** 10.1007/s10880-024-10005-9

**Published:** 2024-02-24

**Authors:** Lara R. LoBrutto, Jared W. Keeley, Natalie D. Dautovich

**Affiliations:** Department of Psychology, Virginia Commonwealth University, 806 West Franklin Street, Richmond, VA 23284 USA

**Keywords:** Somatoform disorders, Medically unexplained symptoms, Pain, Fibromyalgia

## Abstract

Amidst broad changes to the somatic disorder diagnoses, DSM-IV pain disorder was absorbed into DSM-5’s somatic symptom disorder (SSD) as a specifier. However, clinical research testing of its use for the chronic pain population has been limited and its utility remains inconclusive. Using the exemplar of fibromyalgia, this article evaluates the validity, reliability, clinical utility, and acceptability of the SSD pain specifier. The diagnosis appears to have moderate validity but low specificity for the fibromyalgia population. The pain specifier has neither undergone sufficient field testing nor been evaluated for use by medical providers, with available data suggesting low reliability. Further research is needed to establish clinical utility via assessment of differential treatment outcomes. Concerns about social, legal, and economic consequences of classifying pain patients with a mental health diagnosis are outstanding. The current SSD criteria should be used with caution among the fibromyalgia patient population until its application for chronic pain has been further researched.

## Introduction

The controversy around the classification of pain as a psychological diagnosis is long-standing (Hiller et al., 2000; Nordin et al., 2006). Somatization involving pain was classified as a separate diagnosis, pain disorder, in DSM-IV. Revisions to the entire category in DSM-5 led to the absorption of pain disorder into the broader category of somatic symptom disorder (SSD). Although some believe this approach is superior to the previous categorization (Lowe et al., 2022; Wu et al., 2023), there remains questions about its validity, reliability, clinical utility, and acceptability in the diagnosis of patients with serious pain conditions. This narrative review paper seeks to assess the merits and pitfalls of placing fibromyalgia within the SSD diagnosis. Other reviews on the DSM-5 SSD diagnosis (Lowe et al., 2022; Wu et al., 2023) as well as SSD and chronic pain (Katz et al., 2015) have been conducted, but this review is novel in evaluating the intersection of SSD and fibromyalgia. The use of a narrative review allows us to capture all article types, not only empirical works, in order to add to the broader knowledge base on this topic. First, we define fibromyalgia symptomatology, which we use to assess the DSM-5 criteria. Next, we review the diagnostic criteria for DSM-IV pain disorder and DSM-5 somatic symptom disorder and provide a history of somatic symptoms as they pertain to fibromyalgia. Then we review the evidence regarding validity, reliability, clinical utility, and acceptability of using SSD with a chronic pain specifier to diagnose patients with fibromyalgia. Ultimately, we demonstrate that there are substantial limitations to applying DSM-5 SSD to individuals with fibromyalgia in the absence of additional specifications and research.

## Chronic Pain and Fibromyalgia

Chronic pain is defined by the International Association for the Study of Pain as “‘pain without apparent biological value’” that “‘persist[s] beyond the normal tissue healing time… as determined by common medical experience’” (Katz et al., 2015). Chronic pain has a prevalence of 37% in developed countries and is more frequently diagnosed among women and those of lower socioeconomic status (Katz et al., 2015). Although there are many medical diagnoses that may be accompanied by pain, we are focusing the discussion on fibromyalgia for the purposes of this paper, given that there is the greatest amount of evidence around the co-occurrence of this syndrome and SSD (Axelsson et al., 2020; Häuser et al., 2015, 2020; Karlsson et al., 2015; Klaus et al., 2017; Nüesch et al., 2013; Sansone et al., 2004; Wolfe et al., 2013, 2014).

Fibromyalgia, which has a population prevalence of around 2.7%, is characterized by widespread musculoskeletal pain and causes significant disruption in daily life (Axelsson et al., 2020; Häuser et al., 2020; Klaus et al., 2017; Wolfe et al., 2013). The etiology of fibromyalgia is often medically unexplained, which leaves patients suffering from pain without an understanding of its root cause (Klaus et al., 2017). The diagnostic criteria were introduced by the American College of Rheumatology in 1990 and revised in 2010, 2011 and 2016 (Galvez-Sánchez & Reyes Del Paso, 2020; Wolfe et al., 2010, 2016). Per current guidelines, fibromyalgia is diagnosed using cut-off scores on the Widespread Pain Index (WPI) and Symptom Severity Score (SSS), and requires the presence of pain in four out of five regions over a period of at least three months (Häuser et al., 2020; Wolfe et al., 2016). Positive psychological symptoms are not required for diagnosis but cognitive elements, such as catastrophizing and health-related anxiety, are frequently present (Häuser et al., 2020; Sansone et al., 2004). Fibromyalgia is highly comorbid with depression and anxiety, with 53 to 95% of those diagnosed meeting the criteria for one of these disorders (Häuser et al., 2015; Sadr et al., 2023). While questions have been raised in the medical and research community about whether fibromyalgia “actually exists,” it continues to be used in diagnosis (Häuser & Fitzcharles, 2018; Tavel, 2015).

## Diagnostic Criteria

### DSM-IV

In the DSM-IV, pain disorder consisted of five criteria. Criterion A stated that pain is the “predominant focus” and “of sufficient severity to warrant clinical attention.” Criterion B stipulated “clinically significant distress or impairment in social, occupational or other important areas of functioning.” Criterion C specified that “psychological factors are judged to have an important role in the onset, severity, exacerbation, or maintenance of the pain.” Criteria D and E served to differentiate pain disorder from other related disorders (American Psychiatric Association, 1994; Hiller et al., 2000). However, it was found that “pure pain disorder” was not significantly different from those with multiple somatic symptoms based on comparison of coping strategies, hypochondriasis, dysfunctional cognitions, and other mental health comorbidities (Hiller et al., 2000; Nordin et al., 2006). Additionally, there were concerns about the way that DSM-IV defined pain disorder. One concern was with regards to the way that the entire category of somatization disorders was contingent on the presence of medically unexplained symptoms (Haller et al., 2015; Mayou et al., 2005). The second concern was the lack of explanation of the “psychological factors” that might impact the pain presentation (Katz et al., 2015).

Beyond specific issues with the DSM-IV pain disorder category, there were concerns that pain should not be included in the DSM at all and that alternate routes should be sought to prevent the invalidation of physical symptoms. In particular, up to this point, somatization did not have a clear definition, and researchers were identifying it based on symptom count alone (Crombez et al., 2009). Researchers and clinicians have long been wary of the implications of categorizing pain as a mental health diagnosis. Kroenke et al. (2007) recommended that all pain disorders be included on Axis III, with medical diagnoses, and double coded on Axis I as either a discrete disorder (e.g., anxiety or mood disorder) or, if not meeting criteria for an existing diagnosis, listed as “Psychological Factors Affecting a General Medical Condition.” This suggestion lost its relevance when the axial system was removed in the DSM-5. Still, some continue to suggest a different diagnostic approach, opting for the non-specific adjustment disorder over a discrete disorder (Katz et al., 2015).

### DSM-5

**Fig. 1:**
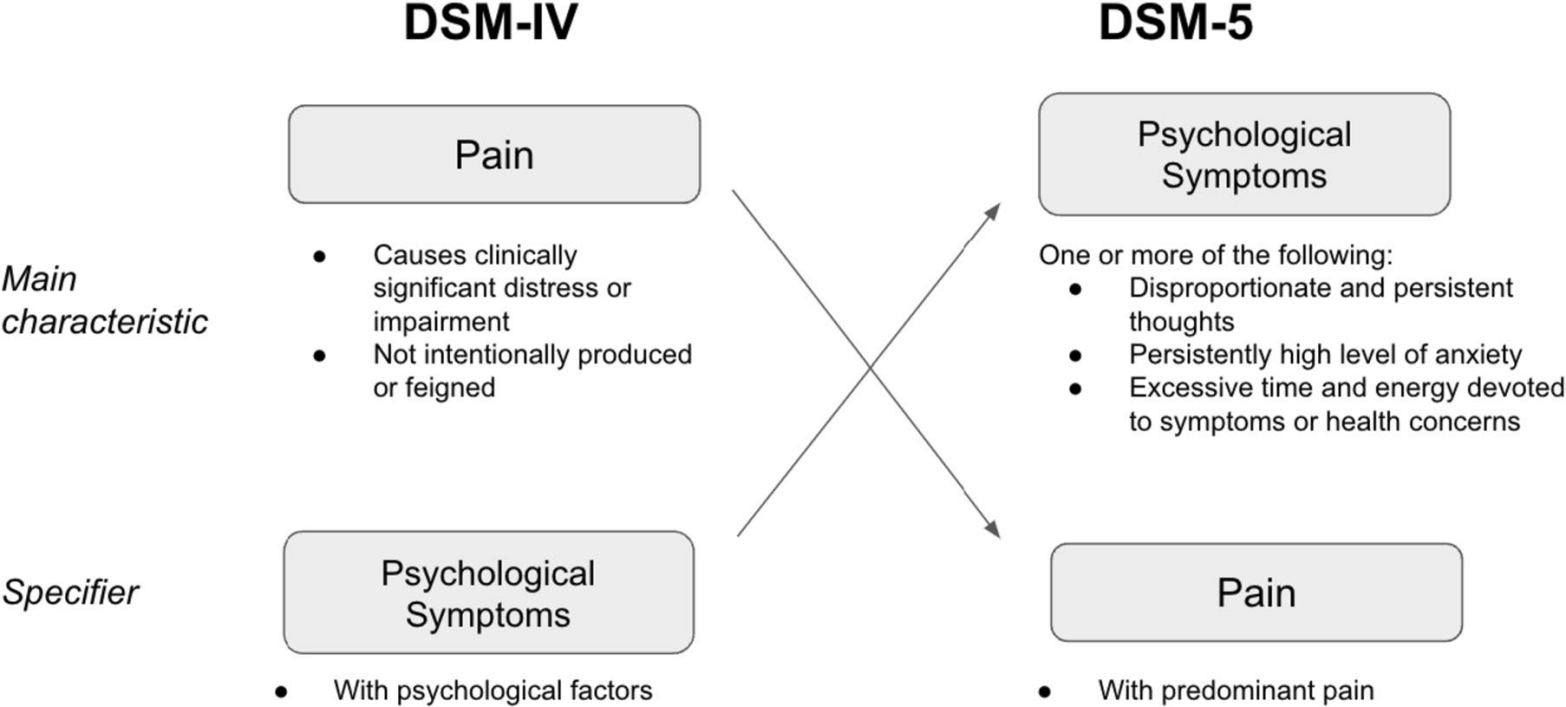
Comparison of diagnostic criteria between DSM-IV and DSM-5

The DSM-5 included major revisions in the area of somatization disorders. Among these changes were the collapsing of diagnostic categories. In the DSM-5, pain disorder is absorbed by the broader diagnosis of somatic symptom disorder, and chronic pain is included as a specifier (American Psychiatric Association, 2013). Somatic symptom disorder contains three criteria. Criterion A is that symptoms are “distressing or result in a significant disruption of daily life.” This is relatively broad as compared with Criterion B of pain disorder, which states that distress is “clinically significant”; impairment, which is specified in the DSM-IV criteria, is not mentioned here. Rather than vaguely alluding to psychological factors as is done in the DSM-IV pain disorder diagnostic category, the DSM-5 notes in Criterion B that “excessive thoughts, feelings, or behaviors” relating to the somatic symptoms must be present in the form of “disproportionate and persistent thoughts about the seriousness of one’s symptoms,” “persistently high level of anxiety about health or symptoms,” and/or “excessive time and energy devoted to these symptoms or health concerns” (American Psychiatric Association, 2013). Determining what constitutes “excessive” relative to their circumstances remains at the clinician’s discretion. Severity level (mild, moderate, or severe) is determined based on cognitive symptoms. Clinicians are asked to rate Criterion B on a scale of 0 to 4, with 0 being “not at all” and 4 being “very much” (Dimsdale et al., 2013). However, the severe specifier may be selected if there are “multiple somatic complaints (or one very severe somatic symptom”; American Psychiatric Association, 2013). A comparison between the DSM-IV and DSM-5 diagnostic criteria can be found in Fig. 1.

## Somatic Symptom Disorder and Fibromyalgia

### Validity

Somatic symptom disorder as applied to the general population has been found to have moderate validity (Löwe et al., 2022). Some authors have recommended modifications to the positive psychological symptoms included in Criterion B; self-concept of weakness and body scanning, for example, have both been identified as factors that would contribute to its discriminant validity by making it more distinct from depressive and anxiety disorders (Wollburg et al., 2013). Predictive validity is supported by findings in a study of somatic inpatients that the diagnosis held at 1-year follow-up (Voigt et al., 2013). Among fibromyalgia patients, prevalence of comorbid SSD ranges from 13.3% to 40% (Axelsson et al., 2020; Häuser et al., 2015, 2020; Sadr et al., 2023; Wolfe et al., 2013). One small study of fibromyalgia patients found an SSD prevalence of 82% but was likely not sufficiently powered (Klaus et al., 2017). In a comparison of fibromyalgia patients and fibromyalgia patients with comorbid SSD, SSD was positively associated with anxiety sensitivity, health anxiety, and reactivity to pain and negatively associated with nonreactivity (Axelsson et al., 2020).

Individuals with SSD also show poorer prognosis (Axelsson et al., 2020). A small cohort study using ecological momentary assessment found cognitions consistent with SSD to be predictive of next-day pain intensity and subjective impairment (Klaus et al., 2017). Patients with SSD may be expected to report higher levels of disability, functional impairment, and healthcare utilization; however, in an fibromyalgia sample, those with comorbid SSD reported higher levels of disability but there were no differences in functional impairment or healthcare utilization (Häuser et al., 2015). Although these findings may suggest a decrease in validity, it is possible that we are seeing a ceiling effect with a population already high in functional impairment and healthcare utilization.

In terms of construct validity, which refers to if the diagnosis relates to other constructs in expected ways, there are concerns that Criterion A is too broadly applicable to an fibromyalgia population, given that between 85 and 97% of fibromyalgia patients meet the criteria, and that Criterion B should require all and not just one of the three symptom presentations for the chronic pain population; in other words, disproportionate and persistent thoughts, high health anxiety, and excessive time and energy spent on the health concerns should all be present if a diagnosis is to be made (Dimsdale et al., 2013; Katz et al., 2015; Wolfe et al., 2013). Information about fibromyalgia that a patient may find online indicates the seriousness of the condition, so it is often difficult to differentiate environmental influences from psychological factors such as catastrophizing, making it all the more important that there is a higher standard for diagnosis when pain is involved (Häuser et al., 2015). Similarly, there are suggestions that the diagnostic criteria require reworking for fibromyalgia patients and that the sensitivity was increased at the expense of specificity, with a false positive rate of about 7%, which may have dangerous implications for those with the already stigmatized condition of medically unexplained pain (Frances & Chapman, 2013; Katz et al., 2015).

### Reliability

The SSD diagnostic criteria are considered to be relatively reliable in the general population. The diagnosis has an interrater reliability of 0.61 and a test–retest reliability of 0.68, both of which fall at the higher end of reliability for DSM diagnoses (Dimsdale et al., 2013). However, for the fibromyalgia population, there are some concerns that the reliability may not hold. Field testing for the new SSD criteria did not explicitly include individuals with chronic pain (Katz et al., 2015). The diagnostic criteria were also not tested with physicians, who are likely to be involved in diagnosis for this population, and the DSM-5 SSD working group stipulates that more research needs to be done with those presenting in medical care settings such as pain medicine (Häuser et al., 2015; Katz et al., 2015). As a result, it is difficult to extrapolate overall reliability to this population. Additionally, interrater reliability was found to be poorer for pain specifiers than for the diagnosis as a whole, perhaps because of the subjectivity of assessing whether pain is out of proportion when an understanding of the pain is based on patient report (Löwe et al., 2022). Nearly a decade has passed since the DSM-5 was released, but there is still a dearth of evidence on the improvement of reliability as the result of familiarity and training.

### Clinical Utility

Overall clinical utility has improved from DSM-IV somatization disorder to DSM-5 somatic symptom disorder, and the new diagnostic category is shown to be among the most useful in DSM-5 field trials (Dimsdale et al., 2013; Löwe et al., 2022). In individuals with somatic symptoms, psychological features were associated with greater levels of disability, suggesting the importance of treatment for this patient population (Rief et al., 2010). In individuals with fibromyalgia, an SSD diagnosis also appears to be somewhat useful. Somatic symptom disorder may be a helpful umbrella diagnosis that more adequately encompasses fibromyalgia and chronic fatigue syndrome (Tavel, 2015). Somatic symptoms preceding a fibromyalgia diagnosis are associated with higher risk factors for fibromyalgia and somatization, suggesting that the DSM-5 SSD criteria may aid in the early diagnosis of fibromyalgia (Creed, 2022). Frequency of psychological symptoms was addressed among clinicians who work with fibromyalgia patients, and 80% of clinicians reported somatic preoccupation, 66% reported hypochondriasis, and 52% reported catastrophizing frequently or very frequently among their patients (Sansone et al., 2004). These findings, in combination with the percentage of individuals meeting an SSD diagnosis in the fibromyalgia population, suggests that differentiating individuals with somatic symptoms might be clinically useful. Similar treatment routes have been used with fibromyalgia and SSD populations. A meta-analysis indicated that cognitive behavioral therapy (CBT) is among the most promising treatments for fibromyalgia for improving primary outcomes of pain and quality of life, and may be useful accompanied by medication for pain and anti-depressants (Nüesch et al., 2013). A randomized control trial with fibromyalgia patients found small but significant decreases in every day stress and increase in life control that was maintained at follow-up but increases in reported pain severity (Katz et al., 2015). It is possible that differentiating those with fibromyalgia from those with fibromyalgia and comorbid SSD might help to identify individuals who might benefit most from CBT. For example, we hypothesize that those without high levels of cognitive symptoms (i.e., no SSD diagnosis) may actually experience increased pain when attention is brought to it, whereas those already experiencing cognitive symptoms such as catastrophizing (i.e., with an SSD diagnosis) may experience relief, although this idea needs to be empirically tested. However, it is also possible that CBT could lead to short-term increases in pain severity for some fibromyalgia patients diagnosed with comorbid SSD, making it important to explore differential treatment needs for those with chronic pain.

### Acceptability

Patient acceptability of somatic disorders has historically been low. Patients report resentment towards the assumption that symptoms are “all in their head “ (Tavel, 2015; Weigel et al., 2020). Although changes to the DSM-5 criteria of somatic disorders is now based on positive symptoms rather than medically unexplained symptoms, which may reduce some stigma, it is often up to therapists to explain the diagnosis in a way that is acceptable to patients (Dimsdale et al., 2013; Weigel et al., 2020). There are concerns that an SSD diagnosis, even as modified in the DSM-5, is based on a male paradigm and could result in negative changes in self-concept among female patients (Frances & Chapman, 2013; Martínez-Lavín, 2021). In the context of fibromyalgia, which generally involves medically unexplained pain, an SSD diagnosis could continue to be harmful. Authors that discuss the comorbid disorders of fibromyalgia and SSD acknowledge that patient acceptability should continue to be explored to ensure that stigma is not perpetuated (Axelsson et al., 2020). Because many individuals experiencing fibromyalgia may not have another medical diagnosis, receiving a mental health diagnosis could lead to cessation of continued testing that could reveal a physical etiology of their pain (Frances & Chapman, 2013). Recommendations have been made to add additional language about developing a clear plan for continued testing, in the case of medically unexplained pain (Katz et al., 2015). There is also some concern about the implications of a mental health diagnosis outside of the medical setting, especially if the diagnosis is unfounded. Legally, individuals who may have received compensation for an injury based on a pain disorder diagnosis in DSM-IV may not be covered based on a SSD diagnosis in DSM-5, and the emphasis on cognitive symptoms might become grounds for blaming the victim (Young, 2010). Additionally, diagnosed individuals may face workplace stigma due to a mental health diagnosis or be denied disability compensation (Frances & Chapman, 2013). Although related systems can make modifications based on the new diagnostic codes, this could take time and potentially cause harm in the interim.

## Conclusion

Although it does appear that placement of chronic pain in the DSM-5 category of somatic symptom disorder is an improvement over DSM-IV pain disorder, this approach is not without concerns for patients with fibromyalgia. Table 1 summarizes the strengths and limitations of the DSM-5 criteria for SSD related to fibromyalgia. A few studies provide evidence that psychological symptoms in fibromyalgia are associated with greater severity and disability, suggesting that placement of disorders related to pain in the DSM is appropriate. However, some authors suggest that the SSD criteria have poor content validity for this population, leading to a high rate of false positives. Interrater reliability is likely poorer for fibromyalgia patients than it is for SSD as a whole, given the clinician’s need to determine whether the psychological symptoms are out of proportion from the physical symptoms, although more research is needed here. There is clinical utility in identifying patients with psychological features and connecting them with treatment, but some evidence suggests an increase in pain as the result of CBT. Furthermore, the acceptability of the diagnosis is low for patients and may have resounding real-world consequences.

**Table 1:** Strengths and limitations of applying the somatic symptom disorder diagnosis to individuals with fibromyalgia

	Validity	Reliability	Clinical utility	Acceptability
Strengths	Next-day and overall functional outcomes differed between SSD groups with and without FMS		May influence choice to use CBT as the treatment modality	
Limitations	SSD criteria have low specificity for an FMS population	Not specifically tested in the chronic pain population Interrater reliability for pain specifiers is lower than that for the overall SSD diagnosis	Differential treatment outcomes remain understudied	Stigma may be increased by the diagnosis, leading to medical, legal, and employment repercussions

Due to the fact that fibromyalgia patients are a population vulnerable to mistreatment in medical spheres, it is important to consider additional guidance for providers and protections for patients. Evidence suggests that SSD is a useful distinction within the fibromyalgia population and will allow patients to be connected with better treatment outcomes, but measures must be taken to avoid harm. It appears that the chronic pain specifier has been tacked on to the SSD diagnosis without accounting for distinctions, which could perpetuate stigma, lead to cessation of medical testing, and create social, workplace, and legal repercussions. Additionally, the diagnosis may be used by both mental healthcare and medical providers, which points to the need for further research and training. Notably, many of the claims of low validity, reliability and acceptability are speculative rather than evidence-based. That said, given the potentially high stakes for individuals with fibromyalgia, there has not been enough testing of the new criteria for use with this population and, consequently, the new DSM-5 criteria should be used with caution by providers until questions of validity, reliability, clinical utility and acceptability are resolved. Future research is needed to assess the utility of the DSM-5 somatic symptom disorder diagnosis for other chronic pain conditions such as migraines and irritable bowel syndrome.

## Data Availability

Not applicable.
